# Resistance to Chronic* Toxoplasma gondii* Infection Induced by a DNA Vaccine Expressing GRA16

**DOI:** 10.1155/2017/1295038

**Published:** 2017-08-10

**Authors:** Ling-Ying Hu, Nian-Zhang Zhang, Fu-Kai Zhang, Meng Wang, Qi Gao, Jin-Lei Wang, Xing-Quan Zhu

**Affiliations:** ^1^State Key Laboratory of Veterinary Etiological Biology, Key Laboratory of Veterinary Parasitology of Gansu Province, Lanzhou Veterinary Research Institute, Chinese Academy of Agricultural Sciences, Lanzhou, Gansu 730046, China; ^2^College of Animal Science, Fujian Agriculture and Forestry University, Fuzhou, Fujian 350002, China; ^3^Hunan Entry-Exit Inspection and Quarantine Bureau, Changsha, Hunan 410004, China; ^4^Jiangsu Co-Innovation Center for the Prevention and Control of Important Animal Infectious Diseases and Zoonoses, Yangzhou University College of Veterinary Medicine, Yangzhou, Jiangsu 225009, China

## Abstract

*Toxoplasma gondii* can infect all warm-blooded animals including human beings.* T. gondii* dense granule protein 16 (TgGRA16) as a crucial virulence factor could modulate the host gene expression. Here, a DNA vaccine expressing TgGRA16 was constructed to explore the protective efficacy against* T. gondii* infection in Kunming mice. The immune responses induced by pVAX-GRA16 were also evaluated. Mice immunized with pVAX-GRA16 could elicit higher levels of specific IgG antibody and strong cellular response compared to those in controls. The DNA vaccination significantly increased the levels of cytokines (IFN-*γ*, IL-2, IL-4, and IL-10) and the percentages of CD4+ and CD8+ T cells in mice. After lethal challenge, mice immunized with pVAX-GRA16 (8.4 ± 0.78 days) did not show a significant longer survival time than that in controls (7.1 ± 0.30 days) (*p* > 0.05). However, in chronic toxoplasmosis model (administration of 10 brain cysts of PRU strain orally), numbers of tissue cysts in mice immunized with pVAX-GRA16 were significantly reduced compared to those in controls (*p* < 0.05) and the rate of reduction could reach 43.89%. The results indicated that the TgGRA16 would be a promising vaccine candidate for further development of effective epitope-based vaccines against chronic* T. gondii* infection in mice.

## 1. Introduction

*Toxoplasma gondii*, an apicomplexan parasite, can infect any nucleated cells of almost all warm-blood mammals including humans and birds [1–3]. Approximately one-third of the global human beings are considered* T. gondii* seropositive [2, 4]. Normally,* T. gondii* infections in immunocompetent people are asymptomatic. However, for people with immunodeficiency, such as AIDS patients and transplant recipients,* T. gondii* infection can lead to encephalitis, ophthalmopathy, or even death [4–6]. Pregnant women primarily infected with* Toxoplasma* could result in miscarriage, ocular complications, and severe neonatal malformations in the fetus [7, 8]. Toxoplasmosis occurring in animals can cause abortion and neonatal loss which can lead to significant economic losses to animal husbandry [9–11].

No drugs are available to eliminate* T. gondii* tissue cysts. Vaccination is considered one of effective options to control and prevent the parasite infection, but until now, no vaccine is suitable for use in humans [12, 13]. Over the last 20 years, encouraging efforts have been made in development of vaccines against* T. gondii* infection [14, 15]. A large number of vaccine candidates were evaluated using DNA vaccinal strategy and most of them were considered to induce effective humoral and cellular immune responses against the cellular pathogen invasion in animal models [16, 17]. However, none [18] of antigens from* T. gondii* could induce more than 80–90% protection against tissue cyst formation.

Dense granule (GRA) is an important secretory organelle in* T. gondii* cytoplasm, and many GRA proteins have been recognized as promising vaccine candidates against the parasite infection [18–21]. Among GRA proteins,* T. gondii* GRA16 (TgGRA16) can be exported beyond the parasitophorous vacuole (PV) to host cell nucleus to modulate the host genome expression [22]. TgGRA16 can bind to the host enzymes (the deubiquitinase HAUSP and PP2A phosphatase) to regulate the ability of p53 protein and the cell cycle in hosts. The p53 protein is a key molecule in control of the inflammation during glucocorticoid responses via regulation of the expression of NF-*κ*B gene [23]. TgGRA16 is also considered a virulent factor for* T. gondii* genotype II strain functioning in the regulation of metabolism and cell cycle. However, it is yet to be known whether TgGRA16 could induce resistance to acute and chronic* T. gondii* infection in the mouse model.

The objectives of this study were to examine the immunogenicity of TgGRA16 using the eukaryotic plasmid pVAX-GRA16 and to estimate its potential protective effect against acute and chronic toxoplasmosis in Kunming mouse models.

## 2. Materials and Methods

### 2.1. Animals

Specific-pathogen-free (SPF) grade inbred Kunming mice (female) of 6–8 weeks of age were purchased from the Center of Laboratory Animals, Lanzhou Institute of Biological Products (Lanzhou, China). All mice were handled in strict accordance with good animal practices required by the Animal Ethics Procedures and Guidelines of the People's Republic of China. The animal study was approved by the Animals Administration and Ethics Committee of Lanzhou Veterinary Research Institute, Chinese Academy of Agricultural Science (Approval Number LVRIAEC2012-011).

### 2.2. Parasites

Two strains of* T. gondii* (RH and PRU) were used in the present study. Tachyzoites of the* T. gondii* RH strain (genotype I) were propagated by a serial intraperitoneal passage in Kunming mice. The tachyzoites were obtained from the peritoneal fluid of mice after centrifuging to remove the cellular debris and then were resuspended in sterile phosphate-buffered saline (PBS). The harvested tachyzoites were also used for the preparation of* Toxoplasma* lysate antigen (TLA) according to our previous description [15]. Tissue cysts of the PRU strain (type II) were maintained in the laboratory by the monthly passage of infective brain homogenate in Kunming mice.

### 2.3. Construction of DNA Vaccines

The TgGRA16 gene was amplified by polymerase chain reaction (PCR) from* T. gondii* genomic DNA with a pair of specific primers (forward primer: 5′-CGGGGTACCATGTATCGAAACCACTCAG-3′ and reverse primer: 5′-CCGGAATTCTCACATCTGATCATTTTTCC-3′) designed according to the reference sequence from ToxoDB (Gene ID: TGME49_208830). The* Kpn* I and* Eco*R I restriction sites were introduced in the primers (underlined).

After sequencing, the PCR products were subcloned into the pVAX I (Invitrogen) through the two restriction sites using T4 DNA ligase and generated recombinant plasmid pVAX-GRA16. The recombinant plasmids pVAX-GRA16 were purified from transformed* E. coli* DH5*α* cells by anion exchange chromatography (EndoFree Plasmid Giga Kit, Qiagen Sciences, MD, USA) following the manufacturer's instructions. The concentration was determined by spectrophotometry at OD260 and OD280. Then the plasmids were dissolved in sterile phosphate-buffered saline (PBS) to a final concentration of 1 *μ*g/*μ*l and stored at −20°C.

### 2.4. Expression of pVAX-GRA16 Plasmids* In Vitro*

HEK293 cells grown on 6-well plates were transfected with recombinant plasmids pVAX-GRA16 using lipofectamine^TM^ 2000 reagent (Invitrogen) according to the manufacturer's instructions. Forty-eight hours after transfection, the expression of pVAX-GRA16* in vitro* was assayed by indirect immunofluorescence assay (IFA) as described previously [24]. The goat anti-*T. gondii* tachyzoites polyclonal antibody (1 : 50 dilution in PBST) and the fluorescein isothiocyanate- (FITC-) labeled rabbit anti-goat IgG antibody (1 : 2000, Sigma, USA) were used as the first antibody and the second antibody and were successively added to each well. As a negative control, the HEK293 cells were transfected with pVAX I.

### 2.5. Immunization and Challenge

A total of 4 groups were performed in the present study (28 mice per group). Mice in different groups were intramuscularly injected with pVAX-GRA16 plasmids, empty pVAX I, and PBS (100 *μ*l/each) 3 times at a 2-week interval (Table 1). The mice that received nothing were used as negative control.

Two weeks after each immunization, blood samples from mice in all groups were collected from the mouse tail vein prior, and then the sera were separated and stored at −20°C. Preimmune serum samples were used as negative control. Two weeks after the third inoculation, 3 mice in each group were sacrificed, and their splenocytes were aseptically harvested for lymphocyte proliferation assay, flow cytometric analysis, and cytokine measurements.

For assessment of protection against acute toxoplasmosis, 10 mice in each group were intraperitoneally (IP) challenged with 10^3^ tachyzoites of the virulent* T. gondii* RH strain 2 weeks after the last immunization. The survival time for each mouse and the percentages of mice survived were recorded until a fatal outcome for all animals. Meanwhile, 6 mice in all groups were inoculated orally with 10 tissue cysts as experimental chronic toxoplasmosis. One month after infection, brains of mice from each group were homogenized in 1 ml PBS. The number of cysts per brain was determined by three samples of 10 *μ*l aliquots of each homogenized brain under an optical microscope.

### 2.6. Determination of Antibodies

The total IgG antibodies in serum samples were determined using SBA Clonotyping System-HRP Kit according to the manufacture's instruction (Southern Biotech Co., Ltd., Birmingham, USA). Briefly, ELISA plates were firstly coated with TLA (15 *μ*g/ml). Twelve hours later, the mouse antisera were added to each well, subsequently with 100 *μ*l of horseradish-peroxidase (HRP) conjugated anti-mouse IgG diluted in 1 : 250. Bound antibody was visualized by adding the substrate solution (PH 4.0) (1.05% citrate substrate buffer; 1.5% ABTS; 0.03% H_2_O_2_) for 20 min. All measurements were performed in triplicate.

### 2.7. Lymphocyte Proliferation Assay by MTs

The harvested splenocytes from 3 mice in each group were plated in triplicate at a density of 5 × 10^5^ cells per well in complete medium (DMEM medium + 10% FCS + 100 U/ml penicillin/streptomycin). The spleen cells of mice from each group were stimulated with TLA (10 *μ*g/ml) as positive controls. Wells that added medium (M) alone served as negative controls. The proliferative activity was measured using MTs method (Promega, USA) after 4 days. The stimulation index (SI) was calculated using the formula OD_570TLA_ : OD_570M_.

### 2.8. Flow Cytometry

Analyses of CD4+ and CD8+ T lymphocytes were performed according to our previous study [25]. The percentages of CD4+ and CD8+ T lymphocytes were determined using flow cytometry with staining by the surface markers including phycoerythrin- (PE-) labeled anti-mouse CD3 (eBioscience), allophycocyanin- (APC-) labeled anti-mouse CD4 (eBioscience), and fluorescein isothiocyanate- (FITC-) labeled anti-mouse CD8 (eBioscience) antibodies. All the samples were analyzed regarding fluorescence profiles on a FACScan flow cytometer (BD Biosciences) by SYSTEM II software (Coulter).

### 2.9. Cytokine Assays

Splenocytes without RBC in 3 mice from each group were stimulated with 10 *μ*g/ml TLA. Culture supernatants were harvested at 24 h for determination of IL-2 and IL-4, 72 h for IL-10, and 96 h for IFN-*γ* and IL-12 (p70) using commercial ELISA kits according to the manufacturer's instructions (Biolegend, USA). The analysis was performed with the data from three independent experiments.

### 2.10. Statistical Analysis

All statistical analyses were performed by SAS procedure (Statistical Analysis System, Version 9.1). The differences of all the data among the 4 groups were analyzed by one-way ANOVA. The results in comparisons between groups were considered different if *p* < 0.05.

## 3. Results

### 3.1. Expression of the Plasmids in HEK293 Cells

The expression of pVAX-GRA16 was identified using IFA. Specific green fluorescence was observed in HEK293 cells transfected with the eukaryotic recombinant plasmid pVAX-GRA16, but not in the controls transfected with the same amount of empty pVAX I.

### 3.2. Evaluation of Antibody against TgGRA16

**Figure 1:**
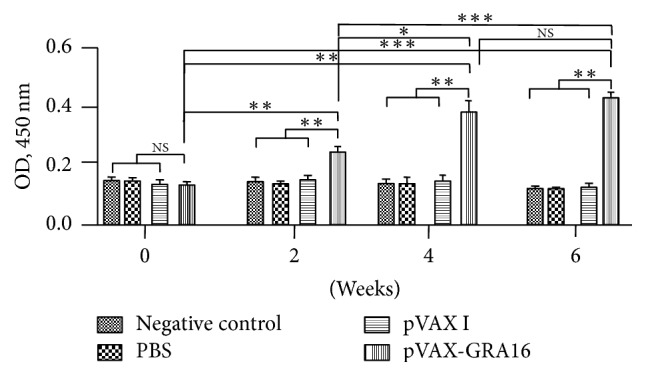
Determination of IgG antibody response induced by DNA immunization with pVAX-GRA16, pVAX I, PBS, and negative controls in the sera of Kunming mice at 0, 2, 4, and 6 weeks by ELISA. Each bar represents the mean OD (±SE, *n* = 3). ^*∗∗∗*^*p* < 0.001, ^*∗∗*^*p* < 0.01, and ^*∗*^*p* < 0.05; NS: not significant.

Levels of IgG antibodies in mice immunized with pVAX-GRA16 were increased with successive DNA vaccination and reached the highest level at 2 weeks after the third booster. The antibody titers in sera collected at 6 weeks were significantly higher than that collected at 0 weeks (*p* < 0.001) and 2 weeks (*p* < 0.001) but showed no significant difference compared to that collected at 4 weeks (*p* > 0.05) (Figure 1). Mice injected with PBS and pVAX I did not generate specific antibodies, which revealed no significant differences compared to those in negative control group (*p* > 0.05).

### 3.3. Evaluation of Splenocyte Proliferation

After coculture of splenocytes with the stimulant, the proliferative response was examined by MTs and calculated as the ratio of the average OD_570TLA_ to the average OD_490M_. The result showed that a vigorous splenocyte proliferation was detected in pVAX-GRA16 immunized mice (*p* < 0.01) compared with splenocytes from mice that received pVAX I, PBS, and nothing (Table 2).

### 3.4. Percentages of CD4+ and CD8+ T Lymphocytes

As shown in Table 2, the ratios of CD4+ T lymphocytes in mice from pVAX-GRA16 immunized group (13.87 ± 1.50) were significantly higher than that in mice from pVAX I group (5.27 ± 1.82), PBS group (3.43 ± 1.53), and negative control (4.17 ± 0.40) (*p* < 0.001). CD8+ T cells in the spleen from mice immunized with pVAX-GRA16 were significantly increased (6.13 ± 0.96) compared to those in pVAX I group (3.60 ± 0.98), PBS group (2.53 ± 1.74), and negative control (4.60 ± 0.95) (*p* < 0.05). But there were no statistically significant differences in both the CD4+ and CD8+ T cells among the three control groups (*p* > 0.05).

### 3.5. Cytokine Production

As shown in Table 3, the Th1-biased cytokines IFN-*γ* (*p* < 0.001) and IL-2 (*p* < 0.001) were significantly increased in spleen cells from mice immunized with pVAX-GRA16 compared to those in mice from pVAX I, PBS, and negative control groups. Meanwhile, splenocytes from pVAX-GRA16 immunized mice also produced higher levels of Th2-biased cytokines IL-4 (*p* < 0.05) and IL-10 (*p* < 0.001) compared to those in all the control groups (Table 3). Levels of various cytokines in spleen cells from mice in pVAX I, PBS, and negative control groups failed to be elicited after being cocultured with stimulants (*p* > 0.05).

### 3.6. Protection against* T. gondii* in Mice

**Figure 2:**
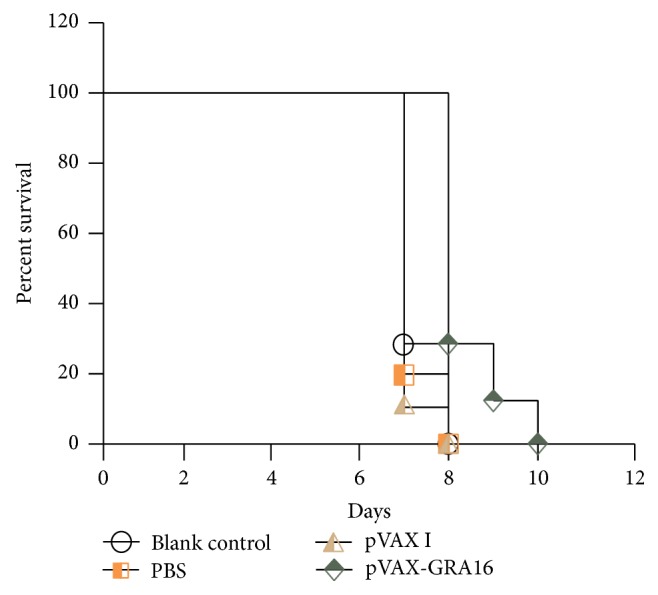
Survival times of mice immunized with pVAX-GRA16, pVAX I, PBS, and nothing followed by challenge with 1 × 10^3^ tachyzoites 2 weeks after the last immunization.

To evaluate whether pVAX-GRA16 can induce effective protection against acute toxoplasmosis, 10 mice from each group were challenged with lethal* T. gondii* tachyzoites of virulent RH strain two weeks after the last immunization. The average survival time of immunized mice (8.4 ± 0.78 days) showed an extension tendency compared to that of the control groups (7.1 ± 0.30 days), but the differences were not significant (*p* > 0.05) (Figure 2).

To determine the protection against* T. gondii* tissue cyst formation in the brain, mice were orally challenged with 10 cysts of the PRU strain. One month later, the brain cyst loadings were assessed. As shown in Table 4, compared to pVAX I, PBS, and negative control groups, immunization with pVAX-GRA16 significantly reduced brain cyst numbers in the immunized mice (*p* < 0.001), with a cyst reduction of 43.89%.

## 4. Discussion

Toxoplasmosis is a zoonotic disease with worldwide distribution [26]. However, no effective and safe medicines are available to control the parasite. It is thus particularly urgent to develop effective vaccines. In recent years, numerous studies have evaluated the protective efficacy of different antigens, such as MIC6 [27], ROP18 [28], NTPase II [29], TrxLp, and ENO2 [30]. Most of them showed partial protection against toxoplasmosis. It is reported that DNA vaccines can induce robust humoral and cellular immune responses [31, 32]. Herein, DNA immunization with TgGRA16 gene induced strong humoral and cellular immune responses, with a slightly longer survival time and a significant brain cyst reduction in mice compared to the controls, suggesting that TgGRA16 would be a potential vaccine candidate.

T cell-mediated immune responses are well known to be resistant against* T. gondii* infection [33]. Particularly, the activity of cytotoxic CD8+ T cells is considered crucial for the destruction of host cells where* T. gondii* niche by the production of IFN-*γ* [34, 35]. Here, immunization of mice with pVAX-GRA16 induced significantly higher levels of CD4+ and CD8+ T lymphocytes, which contributed to a* Toxoplasma*-specific CTL response. The results of enhanced CD4+ and CD8+ T cells in mice immunized with pVAX-GRA16 were consistent with our previous study of DNA vaccination with TgCDPK5 [24].

Productions of IFN-*γ* and IL-2 cytokines could activate macrophage for killing* T. gondii* and also trigger multiple intracellular mechanisms to limit the parasite replication* in vivo* [31, 34]. Here, the significantly high levels of IFN-*γ* and IL-2 cytokines were detected in mice immunized with pVAX-GRA16, which contributed to the decrease of the brain cyst burden compared to that in controls. The pVAX-GRA16 also induced increasing levels of IL-4 and IL-10 in mice, which was consistent with the results of levels of IgG antibody. IL-4 and IL-10 are known as promotion of the proliferation and differentiation of activated B cells [31].

IgG antibodies can opsonize* T. gondii* for phagocytosis and inhibit the parasite reactivation through attaching to the host cell receptors [36]. Our results showed that pVAX-GRA16 could elicit a higher level of IgG antibodies in mice after the third immunization (*p* < 0.001) compared to that in controls, which would contribute to the mildly longer survival time after acute challenge infection.

To assess the protective immunity of the pVAX-GRA16 DNA vaccine, the Kunming mice were challenged intraperitoneally with lethal dosage of tachyzoites of the highly virulent* T. gondii* RH strain and orally with 10 cysts of PRU strain. The results showed that mice immunized with pVAX-GRA16 could slightly prolong the survival time (8.4 ± 0.78 days) and significantly decrease brain cysts (43.89%) compared to those in all the controls, suggesting that TgGRA16 would be a potential vaccine candidate. Further study should be focused on analysis of the epitopes of TgGRA16 by bioinformatics and evaluation of the protective effect of these antigen peptides derived from TgGRA16 against* T. gondii* infection.

In conclusion, the present study showed that DNA immunization with pVAX-GRA16 induced strong humoral and cellular immune responses and reduced the brain cyst formation in mice, indicating that TgGRA16 would be a potential candidate for further development of effective multiepitope vaccines against acute and chronic toxoplasmosis in the mouse model.

## Figures and Tables

**Table 1:** Summary of treatments performed in mice.

Groups	Treatments	Route of administration
I	Nothing	—
II	PBS	Intramuscularly^c^
III	pVAX I^a^	Intramuscularly^c^
IV	pVAX-GRA16^b^	Intramuscularly^c^

^a^Each mouse, 100 *μ*g pVAX I resuspended in 100 *μ*l PBS. ^b^Each mouse, 100 *μ*g pVAX-GRA16 dissolved in 100 *μ*l PBS. ^c^Each mouse, individually injecting the leg muscle 3 times, at a 2-week interval.

**Table 2:** Splenocyte proliferative responses and flow cytometry analysis of T cell subclasses in spleen cells between the immunization and the controls.

Groups	SI (mean ± SD)	CD3+CD4+CD8− (%)	CD3+CD8+CD4− (%)
Blank control	4.44 ± 0.78	4.17 ± 0.40	4.60 ± 0.95
PBS	3.50 ± 0.46	5.27 ± 1.82	3.60 ± 0.98
pVAX I	3.50 ± 0.51	3.43 ± 1.53	2.53 ± 1.74
pVAX-GRA16	5.53 ± 0.26^*∗∗*^	13.87 ± 1.50^*∗*^	6.13 ± 0.96^*∗*^

SI stands for stimulation index; ^*∗*^*p* < 0.05, ^*∗∗*^*p* < 0.01.

**Table 3:** Cytokine productions by splenocytes of immunized Kunming mice after stimulation by Toxoplasma lysate antigen (TLA)^a^.

Groups	Cytokine production (pg/ml)			
	IFN-*γ*	IL-2	IL-4	IL-10
Blank control	169.80 ± 7.69	49.84 ± 3.59	<5	249.65 ± 13.15
PBS	172.40 ± 16.57	47.94 ± 2.94	<5	310.05 ± 37.59
pVAX I	171.36 ± 2.66	51.52 ± 6.07	<5	235.20 ± 36.73
pVAX-GRA16	1313.00 ± 48.33^*∗∗∗*^	270.1 ± 6.20^*∗∗∗*^	8.43 ± 1.58^*∗*^	686.50 ± 87.36^*∗∗∗*^

^a^Splenocytes from mice were obtained two weeks after the last immunization; ^*∗*^*p* < 0.05; ^*∗∗∗*^*p* < 0.001.

**Table 4:** Tissue cyst loadings in the brains of mice after oral challenge with 10 cysts of the *Toxoplasma gondii *PRU strain.

Groups	Number of brain cysts (means ± SD)
Blank control	3366.67 ± 115.47
PBS	3463.33 ± 109.70
pVAX I	3150.00 ± 129.41
pVAX-GRA16	1866.67 ± 152.75^*∗∗∗*^

^*∗∗∗*^Statistically significant difference (*p* < 0.001).
